# Comparative Analysis of Islet Auto-Transplantation Outcome Classification Systems: Evaluating Concordance, Feasibility, and a Data-Driven Approach

**DOI:** 10.3389/ti.2025.14714

**Published:** 2025-07-18

**Authors:** Davide Catarinella, Paola Magistretti, Raffaella Melzi, Alessia Mercalli, Stefano Tentori, Chiara Gremizzi, Vera Paloschi, Simona Sala, Libera Valla, Francesca Aleotti, Sabrina Costa, Francesco De Cobelli, Rossana Caldara, Lorenzo Piemonti

**Affiliations:** ^1^Clinic Unit of Regenerative Medicine and Organ Transplants, IRCCS Ospedale San Raffaele, Milan, Italy; ^2^Diabetes Research Institute, IRCCS Ospedale San Raffaele, Milan, Italy; ^3^Pancreatic Surgery, Pancreas Translational and Clinical Research Center, IRCCS Ospedale San Raffaele, Milan, Italy; ^4^Department of Radiology, Experimental Imaging Center, IRCCS San Raffaele Scientific Institute, Milan, Italy; ^5^ Università Vita-Salute San Raffaele, Milan, Italy

**Keywords:** islet autotransplantation, graft function, classification systems, C-peptide, insulin secretion

## Abstract

A standardized approach to assessing islet autotransplantation outcomes is crucial for evaluating graft function and guiding clinical decisions. This study compares the performance of existing classification systems—Milan, Minneapolis, Chicago, Leicester, Igls, and a novel Data-Driven approach—by evaluating their ability to differentiate transplant outcomes using metabolic and insulin secretion parameters. Our analysis shows strong concordance among Milan, Minneapolis, Chicago, and Igls, primarily due to minor variations in C-peptide thresholds. The Leicester and Data-Driven systems, however, exhibit greater divergence, with the Leicester system simplifying assessment by excluding severe hypoglycemic events and HbA1c, and the Data-Driven approach offering a more dynamic framework without predefined thresholds. Fasting C-peptide levels emerged as a highly reliable predictor of graft function, with the arginine test proving more effective than Mixed Meal Tolerance Test for additional evaluation. The Data-Driven approach provided superior stratification of outcomes, highlighting the importance of residual insulin secretion in metabolic control. These findings suggest that refining classification systems, particularly by considering insulin sensitivity and residual secretion, could enhance long-term patient monitoring and improve our understanding of beta-cell replacement therapies. Further validation across diverse cohorts is essential for broader clinical adoption.

## Graphical Abstract

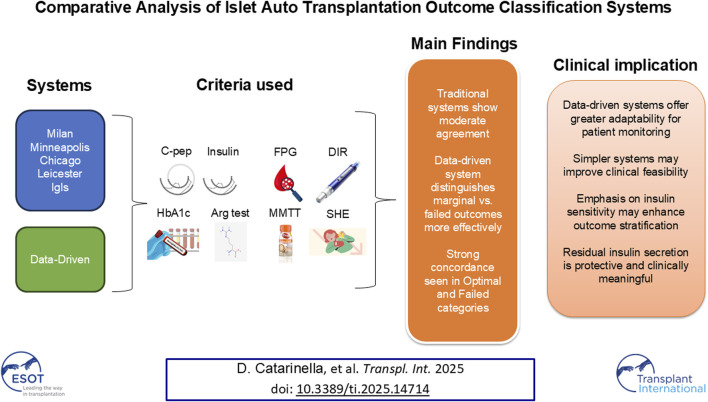

## Introduction

A standardized approach for evaluating the outcomes of beta-cell replacement therapies is essential for enabling comparisons across centers and treatment modalities, including pancreas transplantation, islet transplantation, and stem cell-based interventions [1]. In the context of allotransplantation, a collaborative effort led to the establishment of the Igls criteria, a classification system incorporating key metabolic parameters such as HbA1c levels, frequency of severe hypoglycemic events, insulin requirements, and C-peptide levels [2, 3]. However, the direct application of the Igls criteria presents challenges in the setting of islet autotransplantation (IAT) [4–8]. In IAT, insulin-producing cells from the patient’s pancreas are transplanted, usually after the pancreas is surgically removed [9]. This helps restore insulin production and improve blood sugar control [10]. Unlike allotransplant recipients, patients undergoing pancreatectomy typically do not have pre-existing diabetes and often retain measurable C-peptide secretion prior to the procedure. As a result, the original Igls framework, which evaluates improvements relative to a pre-transplant baseline, may not be suitable for assessing graft function in these patients, since measuring a reduction in insulin requirements or an increase in C-peptide levels relative to pre-pancreatectomy values is not feasible. To address these limitations, several centers have proposed modifications to the Igls criteria to better suit the context of IAT. Notably, institutions in Milan [11], Minneapolis [12], Chicago [13], and Leicester [14] have developed adapted frameworks aimed at more accurately assessing graft function in these patients. Moreover, the original Igls criteria were recently revised to broaden their scope and applicability [4]. These revised approaches consider the unique characteristics of individuals undergoing IAT, ensuring a more appropriate evaluation of post-transplant outcomes. Despite these efforts, a comparative evaluation of the performance of these modified scoring systems remains absent. To bridge this gap, we conducted our study to systematically assess and compare the effectiveness of these adapted criteria in evaluating graft function following IAT. The study sought to determine how well each classification method reflects the functional outcomes of islet transplantation and its capacity to differentiate graft performance using metabolic markers and graft function scores. Additionally, we aimed to develop a Data-Driven classification system to overcome the limitations of arbitrarily defined thresholds traditionally used in graft assessment. By identifying natural clusters within the data, we sought to create a scoring system that more accurately captures the spectrum of graft function and provides an objective, adaptive framework for evaluating post-transplant outcomes.

In some IAT settings, particularly in patients with chronic pancreatitis, outcomes such as pain relief, quality of life, and reduction in narcotic use are central to post-transplant evaluation. However, these aspects are not relevant to our cohort, which—according to the Milan protocol—includes predominantly patients undergoing pancreatectomy for pancreatic neoplasms, high-risk surgical procedures, or postoperative complications, rather than chronic pain. Accordingly, this study focuses exclusively on graft function evaluation through metabolic and insulin secretion parameters.

## Materials and Methods

### Study Objective

The primary aim of this study was to conduct a comparative evaluation of the proposed classification systems for autologous islet transplantation, with the goal of assessing their concordance and their ability to distinguish transplant performance based on the available parameters.

### Study Design

This retrospective observational study included adult patients who underwent total or partial pancreatectomy with IAT at IRCCS Ospedale San Raffaele, Milan, between November 2008 and June 2023 (Clinical Trial. gov: NCT01702051). Data was sourced from a previously published cohort [11]. The study population consisted of patients who underwent pancreatectomy with IAT for indications such as painful chronic pancreatitis, post-surgical pancreatic complications, high-risk pancreaticoduodenectomy, or benign/borderline neoplasms. Eligibility criteria required at least one post-operative follow-up assessment starting from month 1, with sufficient data for the calculation of graft function scores and the availability of at least one standardized stimulation test, either the mixed-meal tolerance test (MMTT) or the arginine stimulation test. Both the MMTT and the arginine stimulation test were used to assess different facets of beta-cell function. The MMTT reflects physiological postprandial insulin secretion in response to mixed nutrients and is therefore more representative of daily metabolic challenges. In contrast, the arginine stimulation test evaluates the maximal insulin secretory response under standardized conditions, making it less susceptible to variations in glucose absorption or gastrointestinal function. This dual approach was employed to capture complementary information on residual islet function across a heterogeneous post-pancreatectomy population. At each available follow-up time point, data were extracted based on the criteria above, enabling the assessment of metabolic and functional parameters. These included fasting plasma glucose, glycated hemoglobin (HbA1c), fasting and stimulated C-peptide levels (measured during both the Arginine and MMTT tests), as well as fasting insulin and proinsulin levels. Beta-cell function was evaluated through the calculation of the area under the curve (AUC) of C-peptide over the first 120 min following the MMTT or arginine test, the insulin peak time during the MMTT, and the acute insulin response to arginine (AIR-arg) during the arginine test. Insulin resistance and beta-cell function indices were derived using the Homeostatic Model Assessment (HOMA), including HOMA-IR for insulin resistance and HOMA-β for beta-cell function, calculated using both C-peptide and insulin levels [15]. All biochemical analyses were performed according to standardized laboratory protocols. The extracted data were used to compare various classification systems for graft function. Continuous glucose monitoring (CGM) data were not included in the present analysis due to the retrospective nature of the study and the lack of standardized CGM use throughout the cohort. During the study period, CGM was not routinely implemented in post-IAT follow-up, particularly in patients without overt diabetes, resulting in incomplete and non-comparable data.

### Mixed Meal Tolerance Test (MMTT)

The MMTT was performed following an overnight fast (≥8 h), using a 250-kcal test meal, consisting of approximately 52% carbohydrates, 11% fats, and 37% proteins. Specifically, the “Boost High Protein Rich Chocolate Balanced Nutritional Drink” (Nestlé Health Science) was used. The drink was consumed within 10 min, and blood samples were collected at baseline (−10 and 0 min), followed by 10, 20, 30, 60, 90, 120, and 180 min after ingestion. The overall beta-cell response to the mixed meal was assessed by calculating the AUC of C-peptide levels over the 120-min test period. The highest C-peptide measurement during the test, referred to as the C-peptide peak, was also recorded.

### Arginine Test

The arginine test was performed following an overnight fast, with insulin therapy suspended prior to the test. A 30-g intravenous bolus of arginine hydrochloride was administered over 30 min. Blood samples for insulin, glucose, and C-peptide concentrations were collected at baseline and at the following time points: 5, 10, 20, 30, 40, 50-, 60-, 90-, and 120-min post-infusion. The acute insulin response to arginine (AIR-arg) was calculated as the incremental AUC of insulin between 0 and 10 min. The overall beta-cell response to the arginine stimulus was assessed by calculating the AUC of C-peptide during the 120-min test period [16, 17].

### Classification Methods

Graft function was assessed using five classification systems, including frameworks developed by institutions in Milan [11], Minneapolis [12], Chicago [13], and Leicester [14], as well as the revised Igls criteria [4]. These classification methods were applied to the study cohort to assess their concordance and ability to distinguish transplant performance. A summary of the classification criteria is provided in Table 1. All systems categorized graft function into four levels. In four of them, the categories were defined as Optimal, Good, Marginal, and Failed. The Leicester classification used a different nomenclature (Good, Partial, Poor, and Failed), which was standardized to align with the four-tier grading of the other systems. Graft function was primarily evaluated based on fasting C-peptide levels, although some classifications allowed for the inclusion of stimulated values. However, given that two different stimulation tests were utilized in this study, and their stimulated C-peptide responses differ, fasting C-peptide was selected as the standard parameter for comparison.

**TABLE 1:** Modified Igls classification after islet Auto-transplantation.

Classification	HbA1c	Severe Hypo episodes (SHE)	Insulin dose	Fasting C-peptide (stimulated)
Igls updates				
Optimal	≤6.5%	None	0 U/kg/d	Any
Good	<7%	None	Any	≥0.2 ng/mL (>0.5 ng/mL)
Marginal	≥7%	≥1	Any	≥0.1 ng/mL (>0.3 ng/mL)
Failed	-	-	Any	<0.1 (≤0.3 ng/m)
Chicago Auto-Igls				
Optimal	≤6.5%	None	0 U/kg/d	>0.5 ng/mL^a^
Good	<7%	None	<0.5 U/kg/day	>0.5 ng/mL^a^
Marginal	≥7%	≥1	≥0.5 U/kg/day	>0.5 ng/mL^a^
Failed		-	-	≤0.5 ng/mL^a^
Minnesota Auto-Igls				
Optimal	≤6.5%	None	None	≥0.2 ng/mL (>0.5 ng/mL)
Good	<7%	None	<0.5 U/kg/d	≥0.2 ng/mL (>0.5 ng/mL)
Marginal	≥7%	≥1	≥0.5 U/kg/d	≥0.2 ng/mL (>0.5 ng/mL)
Failed	-	-	-	<0.2 ng/mL (≤0.5 ng/mL)
Milan Auto-Igls				
Optimal	≤6.5%	None	None	>0.5 ng/mL
Good	<7%	None	<0.5 U/kg/d	>0.5 ng/mL
Marginal	≥7%	≥1	≥0.5 U/kg/d	>0.3 ng/mL
Failed	-	-	-	≤0.3 ng/mL
Leicester Auto-Igls				
Good	–	–	None (up to 5 years)^b^	≥0.2 ng/mL (>0.5 ng/mL)
Partial	–	–	<20 U/d	≥0.2 ng/mL (>0.5 ng/mL)
Poor	–	–	20–40 U/d (within 5 years)^b^	≥0.2 ng/mL (>0.5 ng/mL)
Failed	–	–	–	≤0.5 ng/mL

^a^ The fasting C-peptide value was used.

^b^ The time range was not considered for the calculation.

### Development of a Data-Driven Classification System

To identify natural clusters within the data, an agglomerative hierarchical cluster analysis was performed using three key metabolic variables: HbA1c, fasting C-peptide, and Daily Insulin Requirement (DIR). The optimal number of clusters was determined through dendrogram analysis, which identified four categories (Clusters A, B, C, and D), each exhibiting significant differences in metabolic parameters, as shown in Supplementary Figure S1; Supplementary Table S1. These clusters were ranked in descending order of metabolic outcomes, with Cluster C demonstrating the most favorable profile, followed by A, B, and D. To establish threshold values for each metabolic parameter, Receiver Operating Characteristic (ROC) curve analysis was employed (Supplementary Figure S2). The optimal cut-off points were selected to achieve a specificity of 80%, ensuring reliable differentiation between the clusters. These threshold values for HbA1c, fasting C-peptide, and DIR were subsequently used to assign a score to each variable, with scores ranging from 1 to 4, where higher scores indicated better glucometabolic control. The sum of the scores across the three parameters resulted in a composite glucometabolic score ranging from 3 to 12. The composite glucometabolic score was used to categorize patients into four distinct outcome groups: failure (scores 3–6), marginal control (scores 6–9), good control (scores 9–12), and optimal control (score of 12). This methodology offers a refined and data-driven framework for evaluating glucometabolic regulation, as detailed in Table 2.

**TABLE 2:** Data-Driven classification after islet auto-transplantation.

HbA1c (%)		DIR (U/kg/d)		Fasting C-peptide (ng/mL)	
Value	Score	Value	Score	Value	Score
>7.15	1	>0.57	1	<0.25	1
6.44–7.15	2	0.1–0.57	2	1.52–0.25	2
5.85–6.45	3	0.03–0.1	3	1.89–1.53	3
<5.85	4	<0.03	4	>1.89	4
Glucometabolic outcome classification based on composite score					
Category		Composite score			
Optimal		12			
Good		9 - <12			
Marginal		6 - <9			
Failed		3 - <6			

### Statistical Methods

Data are presented as mean ± standard deviation or median (25th–75th percentile). The clustering process was performed using Euclidean distance to compute pairwise dissimilarities, while Ward’s method was applied for cluster merging, minimizing intra-cluster variance to ensure the formation of homogeneous groups. Threshold values for HbA1c, fasting C-peptide, and DIR for Data-Driven classification were determined through Receiver Operating Characteristic (ROC) curve analysis. The optimal cut-off points were selected to achieve a specificity of 80%. A specificity of 80% was chosen based on common practice in clinical classification studies, where it is widely used as a balanced threshold to ensure clinical reliability while preserving model generalizability. In the context of graft function monitoring, this level of specificity allows for confident identification of impaired metabolic profiles without excessively compromising sensitivity. Agreement between different classification systems was assessed using Fleiss’ Kappa for multiple raters or Cohen’s Kappa for pairwise comparisons. Variability was evaluated using the median coefficient of variation (CVM), calculated as the ratio of the median absolute deviation (MAD) to the median, expressed as a percentage. Differences in dispersion were analyzed using the Brown-Forsythe test. To compare metabolic and secretion parameters across classification groups, the Kruskal-Wallis test was used, followed by Dunn’s multiple comparison test for *post hoc* analysis.

## Result

### Study Population

**FIGURE 1:**
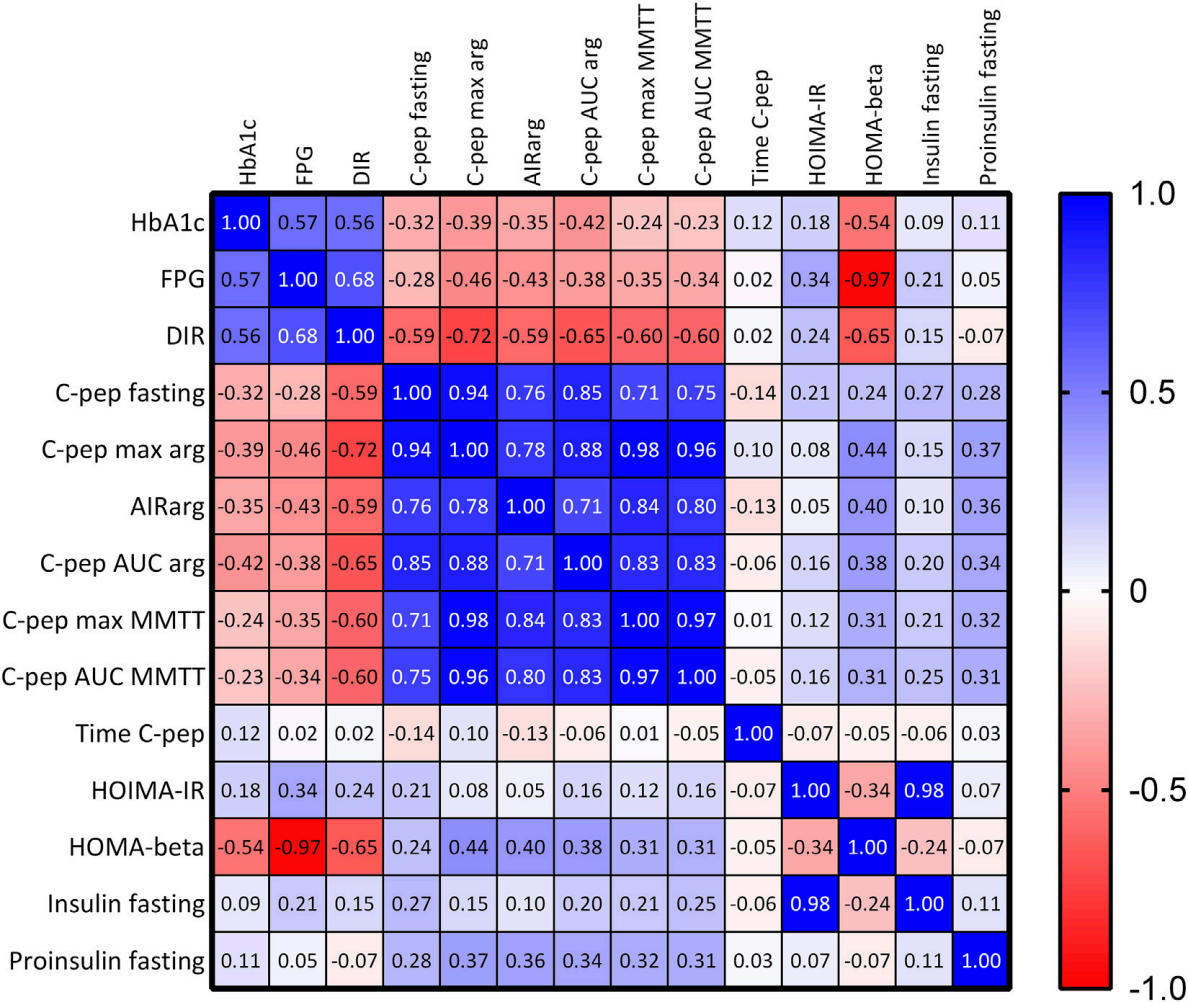
Correlation of Clinical Outcomes in Islet Autotransplantation Patients. The correlation matrix displaying the Spearman R values represented as a heatmap. The color gradient reflects the strength and direction of correlations, with dark blue indicating a strong positive correlation, dark red indicating a strong negative correlation, and lighter shades representing weaker correlations. The numerical values in each cell represent the Spearman correlation coefficient (R), with values close to +1 indicating a strong positive correlation, −1 indicating a strong negative correlation, and 0 indicating no correlation.

The analysis was conducted on data from 88 patients, with a mean age of 59.4 ± 13.8 years, including 40 females. The cohort had a mean BMI of 25.2 ± 4.1 and a mean eGFR of 95.8 ± 30.2. Patients received a median of 1,561 (1,076–2,162) IEQ/kg, with a pre-pancreatectomy fasting C-peptide level of 2.8 ± 1.99 ng/mL and an HbA1c of 5.4% ± 0.58%. Among the patients, 59 had malignant condition, and 72 underwent total or subtotal pancreatectomy. A total of 356 observation points were collected during the follow-up period. Of these, 189 (53%) were gathered within the first-year post-transplant, 117 (33%) between the first and fifth years, and 50 (14%) after 5 years. Among the observation points, 169 (47%) included a MMTT, and 204 (58%) involved an arginine test. Fasting plasma glucose data were available for 342 (96%) of the time points, fasting insulin for 355 (100%), fasting proinsulin for 234 (66%). HbA1c, insulin requirements, and fasting C-peptide levels were available for all patients (100%) as per protocol. A correlation matrix for metabolic outcomes is shown in Figure 1, demonstrating the expected correlation between secretion parameters (resting and stimulated C-peptide) and metabolic outcomes (DIR, FPB, and HbA1c).

### Concordance Between Classification Systems

**FIGURE 2:**
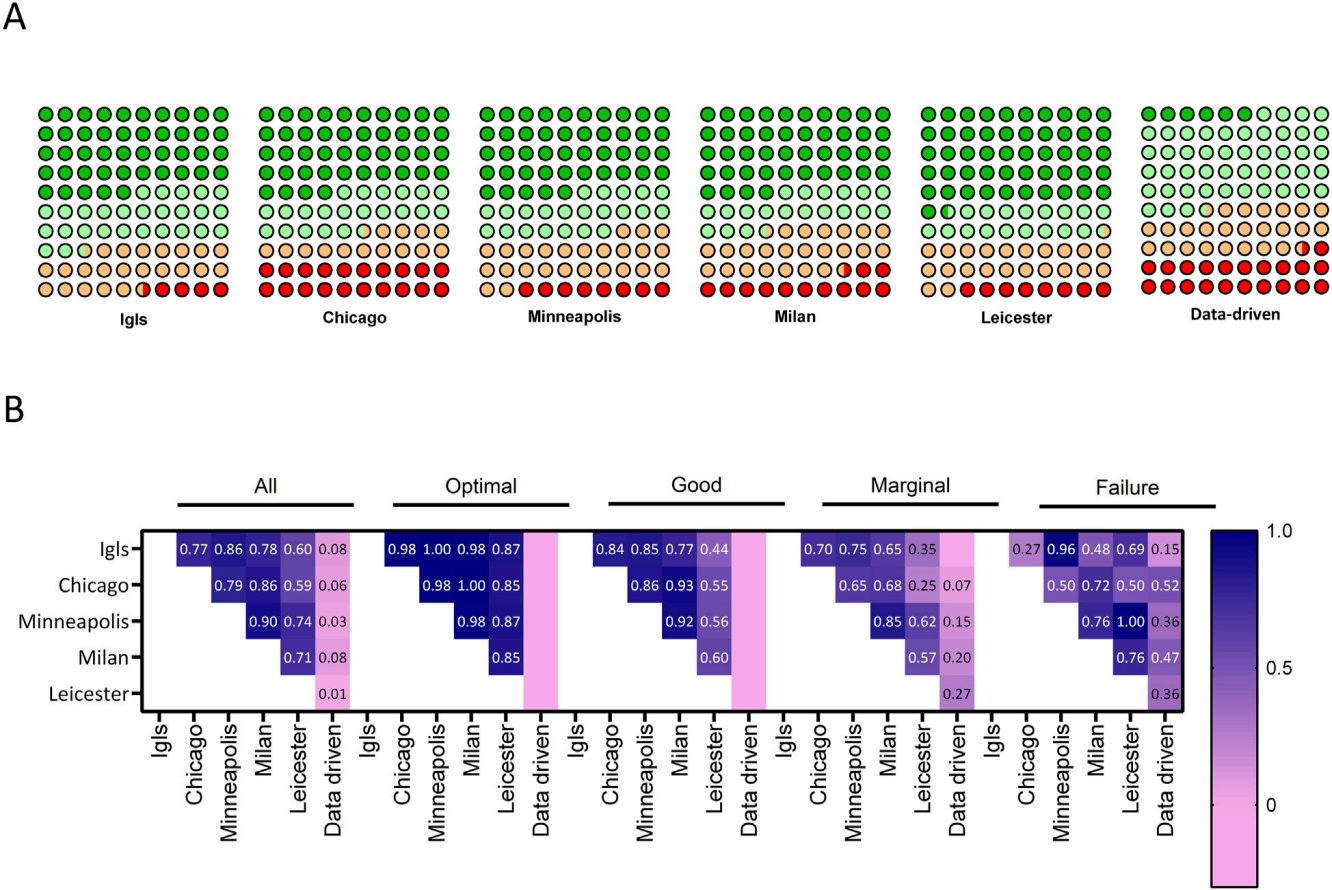
Concordance Between Six Classification Systems for Metabolic Outcomes After Islet Autotransplantation. **(A)** Distribution of patients across four outcome categories. Waffle charts depict the distribution of metabolic outcome categories across six classification systems (Igls, Chicago, Minneapolis, Milan, Leicester, and Data-Driven). Each 10 × 10 grid represents the cohort, with dark green indicating optimal, light green indicating good, orange indicating marginal, and red indicating failed outcomes. **(B)** Heat map of Cohen’s Kappa values. **(B)** Heatmap of Cohen’s Kappa values, showing the level of agreement between the six classification systems. The color gradient represents the strength of concordance, with dark blue indicating strong agreement (Kappa >0.80) and pink indicating poor or no agreement. The values within the grid correspond to the pairwise Cohen’s Kappa coefficients for each classification system comparison.

**FIGURE 3:**
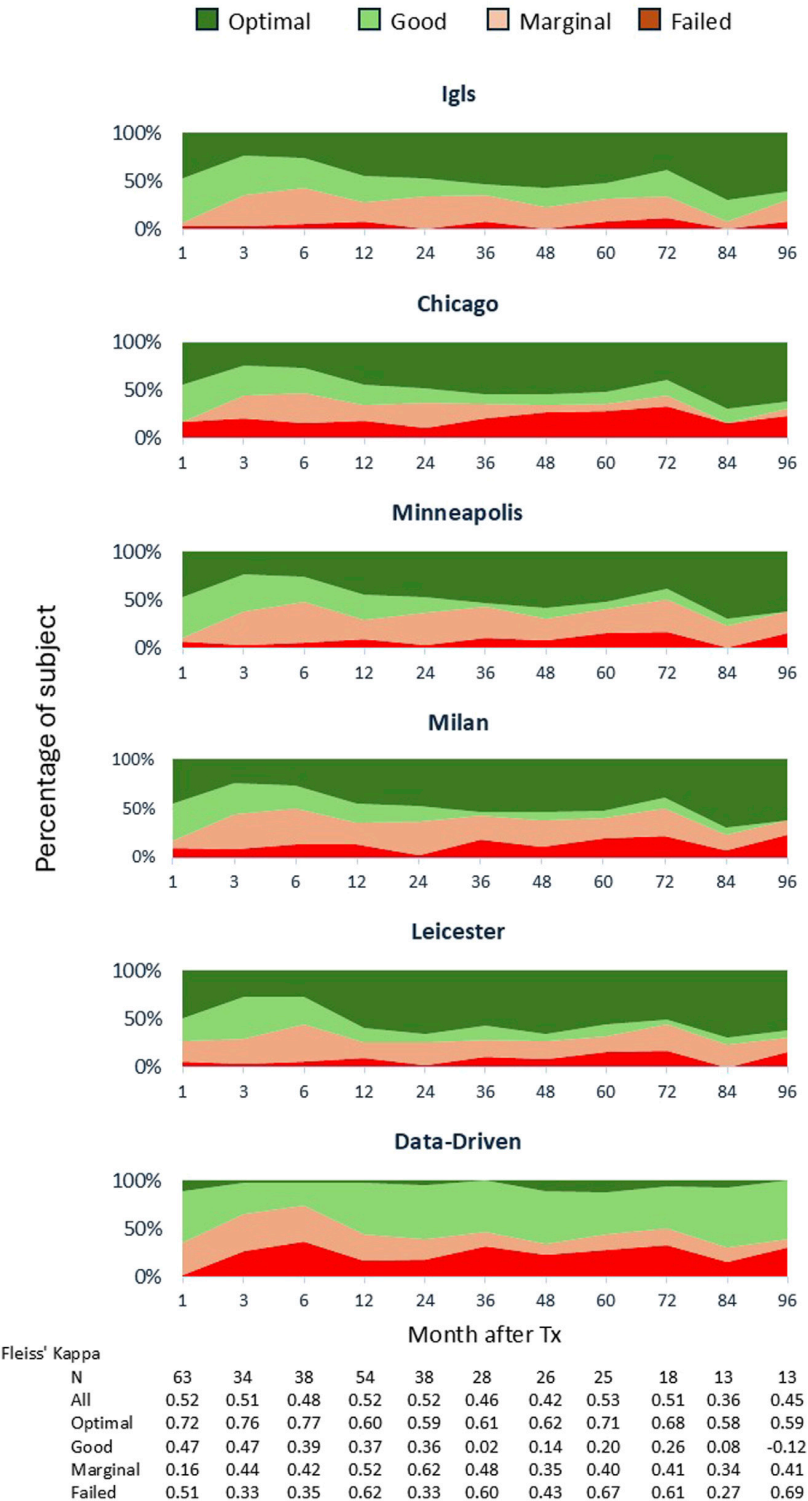
Consistence of Outcome Classification Over Time. The β-cell graft function of 88 IAT cases was assessed and classified as “optimal,” “good,” “marginal,” or “failure” based on the revised criteria (Tables 1, 2). The analysis examined classification consistency at each time point over a 96-month follow-up period. The panels depict β-cell graft function outcomes over time, with Fleiss’ Kappa values reported at the bottom for each time point, evaluated across all categories as well as separately for each category.

A comparative analysis was conducted to evaluate the concordance among six classification systems (Igls, Chicago, Minneapolis, Milan, Leicester, and a Data-Driven approach) in patients undergoing autologous islet transplantation. A visual summary in the form of a comparative schematic that illustrates the key components and thresholds used in each classification system is reported in the Supplementary Table S2. The distribution of patients across the four outcome categories (optimal, good, marginal, and failed) for each classification system is illustrated in Figure 2A. Overall, Fleiss’ Kappa revealed moderate overall agreement among the systems (K = 0.51, p < 0.001). When categorized by outcome, the highest concordance was observed in the optimal (K = 0.68, p < 0.001) and failed groups (K = 0.53, p < 0.001), while lower agreement was found in the marginal (K = 0.45, p < 0.001) and good categories (K = 0.36, p < 0.001), indicating greater variability in classifying intermediate outcomes. This finding was further validated by the analysis of beta cell function over the 8-year follow-up period (Figure 3), which considered each time point and demonstrated that performance remained consistent over time, eliminating the possibility of time-related bias. To evaluate and compare the overall performance of different classification methods, a heat map of Cohen’s Kappa values was generated (Figure 2B), providing a clear visualization of agreement patterns and key trends. Cohen’s Kappa values demonstrated strong agreement among Igls, Chicago, Minneapolis, and Milan classification, with Leicester showing slightly lower concordance. In contrast, the Data-Driven approach exhibited poor agreement with all conventional classifications, underscoring fundamental differences in classification criteria.

### Evaluation of the Consistency and Differentiation Capacity of the Classification Systems Based on Glucose Control Parameters

**FIGURE 4:**
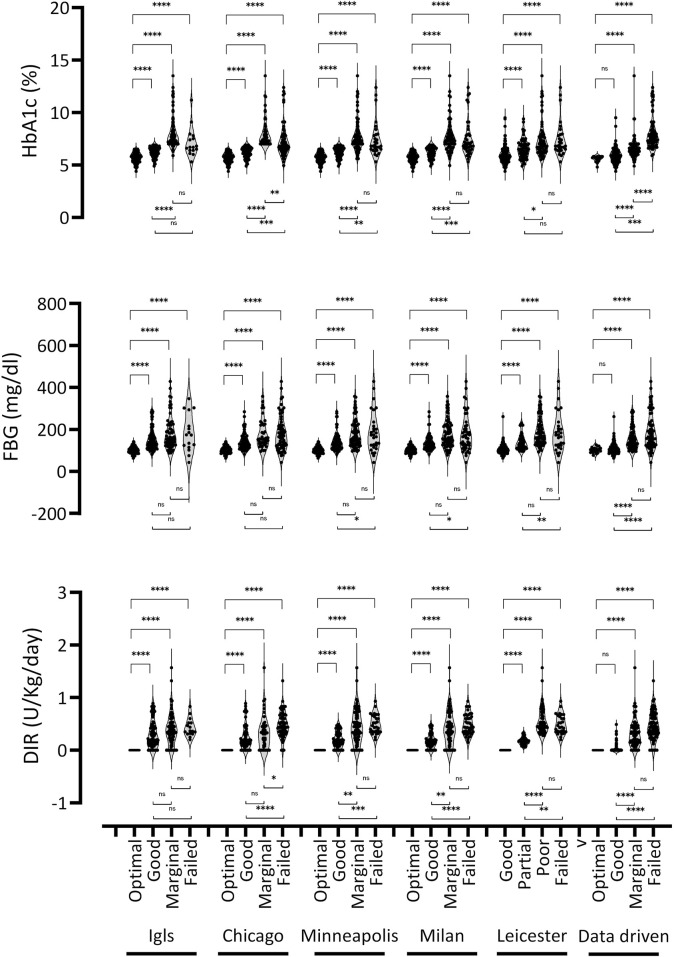
Evaluation of the consistency and differentiation capacity of classification systems based on glucose control parameters. Violin plots depicting the distribution of HbA1c, fasting blood glucose (FBG), and Daily Insulin Requirement (DIR) across the four categories identified by each classification system. Each violin represents a distinct category, with individual data points shown as dots. The width of each violin corresponds to the data density at different values. Statistical differences between categories were assessed using the Kruskal-Wallis test, followed by *post hoc* Dunn’s test. Asterisks indicate statistical significance (*p < 0.05, **p < 0.01, ***p < 0.001, ****p < 0.0001).

To evaluate the consistency of classification systems in identifying actual outcomes, we performed two types of analyses. The first analysis aimed to assess the ability of each classification system to differentiate categories based on glycemic control parameters, including HbA1c, DIR, and fasting glucose. The second analysis focused on determining whether the absolute values of these parameters varied significantly within the same category across different classification systems. The detailed findings are presented in Table 3; Figure 4. When considered collectively, all classification systems significantly differentiate metabolic parameters of glycemic control across the various outcome categories, although with substantial dispersion in values, which increases progressively from the “optimal” to the “failed” category across all classifications. However, *post hoc* analysis provided valuable insights. Most classification systems successfully differentiated between the “optimal” and “good” categories, except for the Data-Driven approach, and between these two and the “failed” category. In contrast, differentiating between the “marginal” and “failed” categories, and to some extent between the “good” and “marginal” categories based on glycemic control, proved challenging. The Data-Driven classification system, however, showed the highest accuracy in making these distinctions. The analysis of absolute values within the same functional category across different classification systems revealed that the values were not always directly comparable. In the “optimal” category, glycemic control parameters were consistently similar across all systems, while in the “Good” and “Marginal” categories, there was greater variability. Overall, the data-driven classification system exhibited the most deviation compared to the others.

**TABLE 3:** Evaluation of the consistency and differentiation capacity of the classification systems based on glucose control parameters.

	HbA1c		FPG		DIR	
	%	CVM (%)	mg/dL	CVM (%)	U/kg/day	CVM (%)
Igls						
Optimal	5.8 (5.3–6)	6.9	100 (91–112)	10.3	0 (0–0)	0
Good	6.4 (5.9–6.7)	4.7	136 (115–169)	17.8	0.18 (0.11–0.32)	83.3
Marginal	7.5 (7.2–8.5)	7.3	156 (126–220)	22.6	0.43 (0.23–0.67)	40.7
Failed	6.6 (6.2–7.7)	9.0	175 (117–274)	31.5	0.44 (0.35–0.65)	23.9
p	<0.0001^a^	<0.0001^b^	<0.0001^a^	<0.0001^b^	<0.0001^a^	<0.0001^b^
Chicago						
Optimal	5.8 (5.3–6.1)	6.9	101 (91–112)	10	0 (0–0)	0
Good	6.4 (5.9–6.6)	4.7	131 (114–160)	16	0.17 (0.10–0.26)	64.7
Marginal	7.7 (7.3–8.8)	7.8	153 (133–222)	20.1	0.38 (0.20–0.59)	46.1
Failed	6.8 (6.3–7.8)	8.8	165 (122–218)	27.7	0.44 (0.35–0.65)	29.5
	<0.0001^a^	<0.0001^b^	<0.0001^a^	<0.0001^b^	<0.0001^a^	<0.0001^b^
Minneapolis						
Optimal	5.8 (5.3–6)	6.9	100 (91–112)	10.3	0 (0–0)	0
Good	6.4 (5.9–6.6)	4.7	131 (112–157)	16	0.18 (0.12–0.22)	60
Marginal	7.3 (7–8.1)	8.2	160 (131–221)	22.5	0.47 (0.38–0.72)	50.5
Failed	6.8 (6.4–7.9)	8.8	174 (120–226)	28.6	0.44 (0.35–0.65)	27.3
	<0.0001^a^	<0.0001^b^	<0.0001^a^	<0.0001^b^	<0.0001^a^	<0.0001^b^
Milan						
Optimal	5.8 (5.3–6.1)	6.9	101 (91–112)	10	0 (0–0)	0
Good	6.4 (5.8–6.6)	4.7	128 (112–152)	13.1	0.17 (0.1–0.26)	41.2
Marginal	7.3 (6.7–7.9)	8.2	159 (129–221)	23	0.38 (0.20–0.59)	47.4
Failed	6.9 (6.4–7.9)	8.7	165 (120–218)	30.3	0.44 (0.35–0.65)	29.5
	<0.0001^a^	<0.0001^b^	<0.0001^a^	<0.0001^b^	<0.0001^a^	<0.0001^b^
Leicester						
Optimal	5.8 (5.3–6.2)	6.9	102 (92–113)	10.5	0 (0–0)	0
Good	6.5 (5.9–7)	8.5	128 (112–154)	14.4	0.18 (0.12–0.22)	25.7
Marginal	6.9 (6.3–7.8)	10.8	169 (137–224)	23.4	0.47 (0.38–0.72)	24.7
Failed	6.8 (6.4–7.9)	8.8	174 (120–226)	28.6	0.44 (0.35–0.65)	27.3
	<0.0001^a^	<0.0001^b^	<0.0001^a^	<0.0001^b^	<0.0001^a^	<0.0001^b^
Data-Driven						
Optimal	5.7 (5.4–5.8)	1.8	102 (92–110)	8.1	0 (0–0)	0
Good	5.9 (5.3–6.2)	6.8	104 (93–117)	11.3	0 (0–0)	0
Marginal	6.5 (6.1–6.9)	6.2	141 (117–180)	20.1	0.28 (0.15–0.44)	50.9
Failed	7.5 (7–8.35)	9.3	165 (126–225)	27	0.43 (0.31–0.65)	37.9
	<0.0001^a^	<0.0001^b^	<0.0001^a^	<0.0001^b^	<0.0001^a^	<0.0001^b^
Among different classification						
	p^a^		p^a^		p^a^	
Optimal	0.0832		0.91		-	
Good	<0.0001^h^		<0.0001^h^		<0.0001^h^	
Marginal	<0.0001^c,e,h^		0.023^i^		<0.0001^c,e,f,l,m^	
Failed	0.0008^m^		0.97		0.85	

^a^ Kruskal-Wallis test.

^b^ Brown-Forsythe test.

Significant at Dunn’s multiple comparisons test: ^c^Igls vs. Leichester; ^d^Chicago vs. Milan;^e^ Chicago vs. Leichester;^f^ Milan vs. Leicester; gIgls vs. Milan;^h^ Data-Driven vs. all others; ^i^ Leicester vs. Data drive; ^l^Minneapolis vs. Data-Driven; ^m^Leicester vs. Data-Driven; ^n^Chicago vs. Data-Driven.

### Evaluation of the Consistency and Differentiation Capacity of the Classification Systems Based on Insulin Secretion Parameters

**FIGURE 5:**
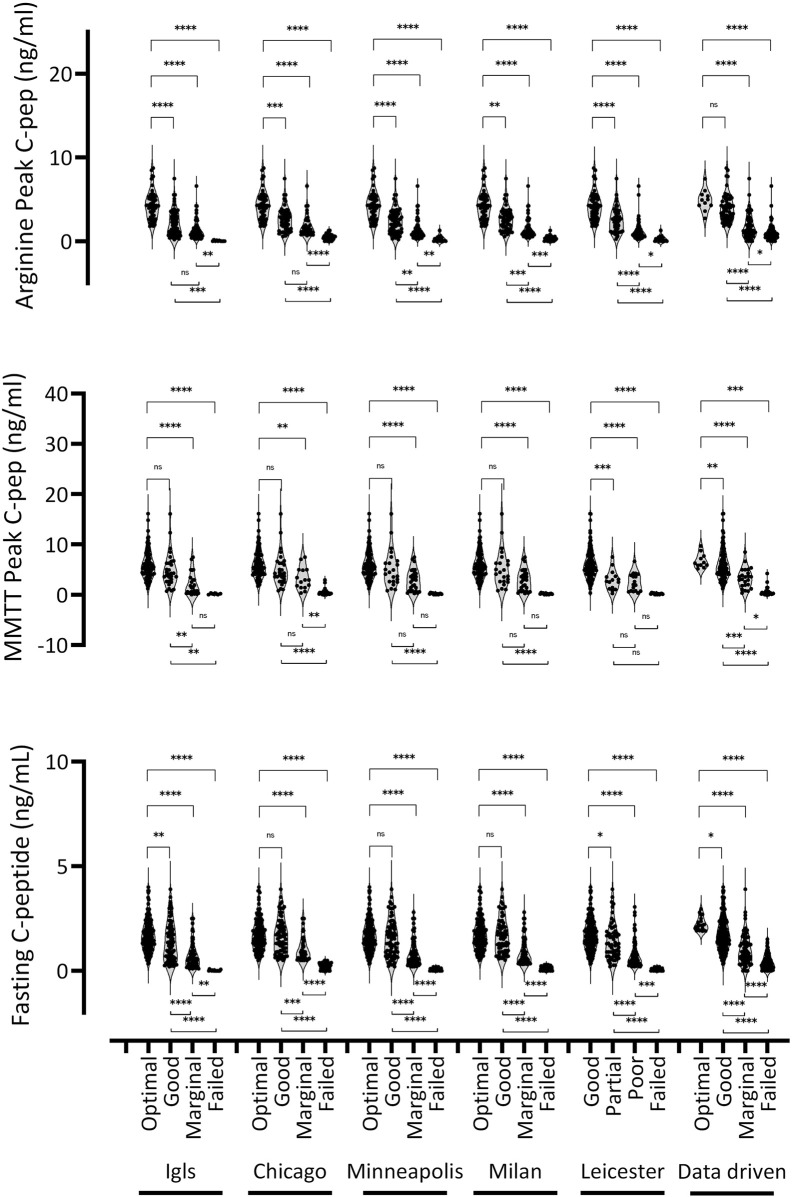
Evaluation of the consistency and differentiation capacity of classification systems based on C peptide secretion parameters. Violin plots illustrate the distribution of fasting C peptide, peak C peptide during an arginine test, and peak C peptide during an MMTT test across the four categories identified by each classification system. Each violin represents a distinct category, with individual data points shown as dots. The width of each violin reflects the data density at various values. Statistical differences between categories were determined using the Kruskal-Wallis test, followed by *post hoc* Dunn’s test. Asterisks denote statistical significance (*p < 0.05, **p < 0.01, ***p < 0.001, ****p < 0.0001).

Fasting and peak C-peptide levels after stimulation were assessed, with detailed findings presented in Table 4; Figure 5. Like glucose control parameters, all classification systems, when considered collectively, significantly differentiated parameters of insulin secretion across the various outcome categories, albeit with substantial variability. However, *post hoc* analysis revealed key differences: unlike glucose control, insulin secretion parameters struggled to distinguish between the “optimal” and “good” categories but effectively differentiated between “good,” “marginal,” and “failed” outcomes. This pattern was partially confirmed by the evaluation of more complex insulin secretion parameters, such as Acute Insulin Response to arginine (AIRarg) and the 2-h C-peptide AUC, although these were less effective than peak C-peptide in differentiating between categories (Supplementary Figure S3; Supplementary Table S3). Additionally, the response to arginine stimulation generally correlated better with classification categories than the response to MMTT. As observed with glucose control parameters, absolute insulin secretion values within the same functional category varied across classification systems, making direct comparisons difficult. In the “optimal” category, insulin secretion parameters were relatively consistent across all classification methods, whereas greater variability was observed in the “good” and “marginal” categories. Notably, the data-driven classification system exhibited the greatest deviation from the others. Further analyses were conducted to assess insulin resistance and β-cell function, including Insulin HOMA-IR, C-peptide HOMA2-%B, fasting insulin, and proinsulin levels (Supplementary Figure S4; Supplementary Table S4). Among these, only C-peptide HOMA2-%B followed the trend of direct insulin secretion parameters, while the others showed less distinct stratification across the “optimal” to “failed” categories. Notably, insulin resistance, as measured by HOMA-IR, was consistently higher in the “good” category compared to the “optimal” category, while no significant differences were observed among the other groups.

**TABLE 4:** Evaluation of the consistency and differentiation capacity of the classification systems based on insulin secretion parameters.

	Fasting C peptide		Arginine peak C-peptide		MMTT peak C-peptide	
	ng/mL	CVM (%)	ng/mL	CVM (%)	ng/mL	CVM (%)
Igls						
Optimal	1.6 (1.2–2.1)	29	4.25 (2.9–5.2)	26	5.4 (4.1–7.7)	25
Good	1.3 (0.6–2)	54	1.8 (0.8–3)	57	4.2 (2.7–6.4)	41
Marginal	0.6 (0.3–0.9)	55	1.02 (0.6–1.6)	38	1.1 (0.3–3.1)	77
Failed	0 (0–0)	0	0 (0–0.1)	0	0.2 (0–0.3)	55
p	<0.0001^a^	<0.0001^b^	<0.0001^a^	<0.0001^b^	<0.0001^a^	0.0778^b^
Chicago						
Optimal	1.6 (1.2–2.1)	27	4.2 (2.9–5.2)	26	5.5 (4.2–7.8)	26
Good	1.6 (0.95–2.1)	40	2.6 (1.4–3.4)	39	4.3 (2.9–6.5)	40
Marginal	0.85 (0.6–1.3)	34	1.4 (1–2.4)	33	2.9 (1.4–5)	52
Failed	0.25 (0.1–0.35)	44	0.5 (0.3–0.7)	42	0.3 (0.2–0.7)	55
	<0.0001^a^	<0.0001^b^	<0.0001^a^	<0.0001^b^	<0.0001^a^	0.0038
Minneapolis						
Optimal	1.6 (0.2–2.1)	29	4.3 (2.9–5.2)	26	5.4 (4.1–7.7)	25
Good	1.3 (0.7–2.1)	48	2.3 (1.12–3.3)	47	4.5 (2.6–6.7)	41
Marginal	0.6 (0.4–1.1)	44	0.98 (0.7–1.4)	39	2.9 (0.8–4.3)	67
Failed	0.07 (0–0.2)	86	0.15 (0–0.5)	93	0.2 (0–0.3)	43
	<0.0001^a^	<0.0001^b^	<0.0001^a^	<0.0001^b^	<0.0001^a^	0.0019
Milan						
Optimal	1.6 (1.2–2.1)	27	4.2 (2.9–5.2)	26	5.4 (4.1–7.7)	25
Good	1.6 (0.95–2.2)	41	2.6 (1.5–3.4)	35	4.5 (2.6 6.7)	41
Marginal	0.6 (0.4–1.2)	39	1.1 (0.8–1.6)	32	2.9 (0.8–4.3)	67
Failed	0.17 (0.03–0.2)	53	0.34 (0.1–0.5)	49	0.2 (0–0.3)	43
	<0.0001^a^	<0.0001^b^	<0.0001^a^	<0.0001^b^	<0.0001^a^	0.0019
Leicester						
Optimal	1.6 (1.2–2.2)	29	4.21 (3.-5.1)	24	5.4 (4.2–7.6)	26
Good	1.3 (0.7–1.8)	44	2.44 (1.1–3.4)	47	2.7 (1.2–3.7)	48
Marginal	0.5 (0.3–0.9)	42	0.9 (0.6–1.4)	41	2 (0.7–4)	72
Failed	0.1 (0–0.2)	86	0.1 (0–0.5)	93	0.2 (0–0.3)	43
	<0.0001^a^	<0.0001^b^	<0.0001^a^	<0.0001^b^	<0.0001^a^	0.033
Data-Driven						
Optimal	2.2 (1.9–2.4)	11	4.9 (4.3–5.6)	13	6.3 (5.9–7.7)	14
Good	1.6 (1.2–2.2)	31	3.8 (2.7–5.1)	28	5.4 (4.1–7.5)	27
Marginal	0.9 (0.5–1.4)	48	1.2 (0.8–2.5)	58	3.3 (1.8–4.8)	44
Failed	0.3 (0.2–0.6)	60	0.8 (1.3–0.4)	49	0.3 (0.2–0.8)	69
	<0.0001^a^	<0.0001^b^	<0.0001^a^	0.0069^b^	<0.0001^a^	0.0008^b^
Among different classification						
	p^a^		p^a^		p^a^	
Optimal	0.0030^h^		0.7346		0.6832	
Good	0.007^d,i^		<0.0001^h^		0.0007^i^	
Marginal	<0.0001^e,i,m,n,o^		0.0023 ^f,o^		0.0993	
Failed	<0.0001^e,g,i,l,m,n,,o,p^		<0.0001^n,h^		0.0687	

^a^ Kruskal-Wallis test.

^b^ Brown-Forsythe test.

Significant at Dunn’s multiple comparisons test:^c^ Igls vs. Leichester;^d^ Chicago vs. Milan;^e^ Chicago vs. Leichester;^f^ Milan vs. Leicester; ^g^Igls vs. Milan;^h^ Data-Driven vs. all others;^i^ Leicester vs. Data drive;^l^Minneapolis vs. Data-Driven;^m^Igls vs. Data-Driven; ^n^ Igls vs. Chicago; ^o^ Chicago vs. Minneapoli; ^p^ Milan vs. Data-Driven.

## Discussion

The comparative evaluation of classification systems for IAT presented in this study underscores the value of multiple existing frameworks while also highlighting key differences that may influence their practical utility. The choice of one classification system over another appears to be dictated less by its intrinsic ability to differentiate metabolic outcomes and more by considerations of feasibility, simplicity, and the number of parameters required for implementation [18]. This aspect is particularly relevant in the clinical setting, where the complexity of obtaining certain metabolic parameters can impact the widespread applicability of a given classification method [10].

One of the most notable findings of this study is the advantage conferred by classification systems that exclude severe hypoglycemic events (SHE) as a criterion, such as the Data-Driven approach and the Leicester system. SHE remains one of the most challenging variables to standardize, as its assessment relies heavily on patient-reported data, which can be prone to subjectivity and recall bias. Nonetheless, the clinical relevance of preventing SHEs should not be overlooked, particularly in insulin-treated patients following total pancreatectomy. Importantly, the impact of SHEs on long-term outcomes has been significantly reduced in recent years with the introduction of advanced diabetes technologies, including CGM, insulin pumps, and hybrid closed-loop systems. These tools have markedly improved hypoglycemia detection and prevention, which may partly justify the omission of SHEs from simplified classification systems in selected clinical contexts. The Leicester system further reduces complexity by not requiring glycated hemoglobin as a mandatory parameter, thereby increasing its practicality in real-world applications. These considerations suggest that classification systems prioritizing feasibility and ease of calculation may be more suitable for routine clinical use, particularly in settings with limited resources or less frequent metabolic monitoring.

From a conceptual standpoint, the strong correlation observed among the Milan, Minnesota, Chicago, and Igls classifications is not surprising, given that they primarily differ in their thresholds for fasting and stimulated C-peptide levels. This convergence reinforces the robustness of C-peptide as a central biomarker in graft function assessment [19]. However, an interesting observation emerged when comparing the “good” and “optimal” outcome categories across all classifications: while these groups exhibited no significant differences in insulin secretion, they did show distinct variations in glucose control. This suggests that factors beyond insulin production—such as insulin sensitivity—may play a critical role in differentiating these groups. The finding that HOMA-IR was significantly higher in the “good” group than in the “optimal” group supports the hypothesis that differences in insulin resistance, rather than secretion capacity, may contribute to variations in glycemic control. This insight is particularly relevant in the broader context of beta-cell replacement and diabetes management, where therapeutic strategies often focus on preserving or enhancing residual insulin secretion without always accounting for the impact of insulin sensitivity on metabolic outcomes.

Equally significant is the differentiation between the “marginal” and “failed” categories. Unlike the distinction between “good” and “optimal,” which appears to be driven by insulin resistance, the primary factor separating “marginal” from “failed” function is the presence of residual insulin secretion. This observation aligns with existing literature on beta-cell replacement therapies, where even minimal levels of residual C-peptide secretion have been associated with protection against severe hypoglycemia and reduced progression of microvascular complications. Fasting C-peptide values ranging from 0.09 to 0.2 ng/mL have been reported as sufficient for these protective effects, reinforcing the clinical significance of residual beta-cell function. This finding has broader implications beyond IAT, extending to the field of type 1 diabetes treatment, where preservation of C-peptide at disease onset is increasingly recognized as a therapeutic goal [20–24]. In the context of IAT, where classification serves primarily as a descriptive tool rather than a determinant of therapeutic interventions, understanding the long-term impact of residual insulin secretion on patient health may provide valuable insights into post-transplant metabolic outcomes.

Another critical consideration is the role of fasting *versus* stimulated C-peptide in classification. Our findings suggest that fasting C-peptide alone is highly informative and may be sufficient for functional assessment in many cases, reducing the necessity for more complex stimulation tests. However, when stimulation is required, the Arginine test appears to provide better differentiation than the MMTT. This is a key observation, as the arginine test is generally easier to standardize and less time-consuming than a full MMTT, making it a more practical choice for post-transplant metabolic evaluations.

A particularly intriguing outcome of this study is the performance of the Data-Driven classification system, which avoids predefined threshold values by leveraging natural clustering of metabolic parameters. This methodology offers a flexible and adaptive framework that may better capture the heterogeneity of post-transplant metabolic function. While the Data-Driven system was more restrictive in defining “optimal” outcomes compared to conventional classifications, it demonstrated superior granularity in distinguishing between different levels of graft function. This suggests that data-driven approaches could serve as powerful tools for refining outcome assessments in IAT. However, further validation in larger and more diverse cohorts is necessary before widespread adoption can be considered.

Despite its strengths, this study has several limitations that should be acknowledged. First, the analysis was conducted in a single-center cohort, which may limit generalizability to other institutions with different patient populations, surgical techniques, or follow-up protocols. Additionally, while the study incorporated many metabolic parameters, it did not evaluate long-term clinical outcomes such as quality of life, diabetes-related complications, or the durability of graft function beyond 8 years. Future studies should aim to address these gaps by integrating patient-reported outcomes and long-term metabolic trajectories. Finally, while the Data-Driven classification demonstrated promising results, its reliance on retrospective data raises questions about its applicability in prospective clinical settings. Further research is needed to determine whether this approach can be successfully implemented in real-time decision-making.

This study provides a comprehensive evaluation of existing classification systems for IAT and introduces a novel Data-Driven approach that may offer advantages in terms of adaptability and differentiation. The findings highlight the strengths and limitations of different frameworks, emphasizing that the choice of a classification system should consider both scientific validity and practical feasibility. The insights gained from this analysis contribute to a broader understanding of beta-cell function assessment and may inform future refinements in transplantation and diabetes care. Ultimately, continued research and collaborative efforts will be essential to optimize graft function evaluation and improve long-term outcomes for patients undergoing IAT.

## Data Availability

The raw data supporting the conclusions of this article will be made available by the authors, without undue reservation.

## References

[1] Piemonti L de Koning EJP Berney T Odorico JS Markmann JF Stock PG Defining Outcomes for Beta Cell Replacement Therapy: A Work in Progress. Diabetologia (2018) 61(6):1273–6. Epub 20180306. 10.1007/s00125-018-4588-0 29511779 PMC6467463

[2] Rickels MR Stock PG Koning EJP Piemonti L Pratschke J Alejandro R Defining Outcomes for Β-Cell Replacement Therapy in the Treatment of Diabetes: A Consensus Report on the Igls Criteria from the Ipita/Epita Opinion Leaders Workshop. Transplantation (2018) 102:1479–86. 10.1097/TP.0000000000002158 29528967 PMC6408213

[3] Rickels MR Stock PG de Koning EJP Piemonti L Pratschke J Alejandro R Defining Outcomes for β-Cell Replacement Therapy in the Treatment of Diabetes: A Consensus Report on the Igls Criteria from the IPITA/EPITA Opinion Leaders Workshop. Transpl Int (2018) 31(4):343–352. 10.1111/tri.13138 29453879 PMC5867272

[4] Landstra CP Andres A Chetboun M Conte C Kelly Y Berney T Examination of the Igls Criteria for Defining Functional Outcomes of Β-Cell Replacement Therapy: Ipita Symposium Report. The J Clin Endocrinol & Metab (2021) 106(10):3049–59. 10.1210/clinem/dgab386 34061967 PMC8571711

[5] Bellin MD. Success with Islet Autotransplantation for Pancreatic Neoplasia Using a Careful Approach. Transplantation (2024) 108(9):1830–1. Epub 20240521. 10.1097/tp.0000000000005050 38771100

[6] Balzano G Zerbi A Aleotti F Capretti G Melzi R Pecorelli N Total Pancreatectomy With Islet Autotransplantation as an Alternative to High-Risk Pancreatojejunostomy After Pancreaticoduodenectomy: A Prospective Randomized Trial. Ann Surg (2023) 277(6):894–903. Epub 20220930. 10.1097/SLA.0000000000005713 36177837 PMC10174105

[7] Balzano G Maffi P Nano R Mercalli A Melzi R Aleotti F Autologous Islet Transplantation in Patients Requiring Pancreatectomy: A Broader Spectrum of Indications Beyond Chronic Pancreatitis. Am J Transpl (2016) 16(6):1812–26. Epub 2015/12/24. 10.1111/ajt.13656 26695701

[8] Balzano G Piemonti L. Autologous Islet Transplantation in Patients Requiring Pancreatectomy for Neoplasm. Curr Diab Rep (2014) 14(8):512. 10.1007/s11892-014-0512-2 24915889

[9] Chen ME Desai CS. Current Practices in Islet Cell Autotransplantation. Expert Rev Endocrinol Metab (2023) 18(5):419–25. Epub 20230907. 10.1080/17446651.2023.2256407 37680038

[10] Bellin MD Eaton A Ramanathan K Beilman G Downs E Schwarzenberg SJ Long-Term Islet Graft Functional Assessment in More than 500 Patients Undergoing Total Pancreatectomy with Intraportal Islet Autotransplantation. J Am Coll Surg (2025) 240:405–13. Epub 20250122. 10.1097/xcs.0000000000001294 39840847

[11] Piemonti L Melzi R Aleotti F Capretti G Nano R Mercalli A Autologous Pancreatic Islet Cell Transplantation Following Pancreatectomy for Pancreas Diseases Other than Chronic Pancreatitis: A 15-Y Study of the Milan Protocol. Transplantation (2024) 108:1962–75. Epub 20240419. 10.1097/TP.0000000000005037 38637923 PMC11335085

[12] McEachron KR Yang Y Hodges JS Beilman GJ Kirchner VA Pruett TL Performance of Modified Igls Criteria to Evaluate Islet Autograft Function After Total Pancreatectomy with Islet Autotransplantation–a Retrospective Study. Transpl Int (2021) 34(1):87–96. 10.1111/tri.13762 33020957 PMC7913469

[13] Gołębiewska JE Bachul PJ Fillman N Basto L Kijek MR Gołąb K Assessment of Simple Indices Based on a Single Fasting Blood Sample as a Tool to Estimate beta-cell Function After Total Pancreatectomy with Islet Autotransplantation - A Prospective Study. Transpl Int (2019) 32(3):280–90. 10.1111/tri.13364 30353611

[14] Pollard CA Chung WY Garcea G Dennison AR. Assessment of Long-Term Graft Function Following Total Pancreatectomy and Autologous Islet Transplantation: The Leicester Experience. Hepatobiliary Surg Nutr (2023) 12(5):682–91. Epub 20220615. 10.21037/hbsn-21-558 37886183 PMC10598318

[15] Wallace TM Levy JC Matthews DR. Use and Abuse of Homa Modeling. Diabetes Care (2004) 27(6):1487–95. 10.2337/diacare.27.6.1487 15161807

[16] Caumo A Maffi P Nano R Luzi L Hilbrands R Gillard P Comparative Evaluation of Simple Indices of Graft Function After Islet Transplantation. Transplantation (2011) 92(7):815–21. 10.1097/TP.0b013e31822ca79b 21836536

[17] Piatti PM Pontiroli AE Caumo A Santambrogio G Monti LD Costa S Hyperinsulinemia Decreases Second-phase but Not First-phase Arginine-Induced Insulin Release in Humans. Diabetes (1994) 43(9):1157–63. 10.2337/diab.43.9.1157 7915241

[18] Gnikpingo T Benson S Hodges JS Downs E Cook M Schwarzenberg SJ Islet Graft Function by Mixed Meal Tolerance Testing Is Sustained Over 4 Years in Young Children Undergoing Total Pancreatectomy With Islet Autotransplantation. Clin Transpl (2023) 37(12):e15106. Epub 20230831. 10.1111/ctr.15106 PMC10841198 37650380

[19] Taylor GS Shaw AC Smith K Wason J McDonald TJ Oram RA Capturing the Real-World Benefit of Residual Β-Cell Function During Clinically Important Time-Periods in Established Type 1 Diabetes. Diabet Med (2022) 39(5):e14814. Epub 20220310. 10.1111/dme.14814 35181926 PMC9311680

[20] Lachin JM McGee P Palmer JP, DCCT/EDIC Research Group. Impact of C-Peptide Preservation on Metabolic and Clinical Outcomes in the Diabetes Control and Complications Trial. Diabetes (2014) 63(2):739–48. Epub 2013/10/04. 10.2337/db13-0881 24089509 PMC3900540

[21] Davis AK DuBose SN Haller MJ Miller KM DiMeglio LA Bethin KE Prevalence of Detectable C-Peptide According to Age at Diagnosis and Duration of Type 1 Diabetes. Diabetes Care (2015) 38(3):476–81. 10.2337/dc14-1952 25519448

[22] Kuhtreiber WM Washer SL Hsu E Zhao M Reinhold P 3rd Burger D Low Levels of C-Peptide Have Clinical Significance for Established Type 1 Diabetes. Diabet Med (2015) 32(10):1346–53. Epub 2015/07/15. 10.1111/dme.12850 26172028 PMC4578991

[23] Ludvigsson J. The Clinical Potential of Low-Level C-Peptide Secretion. Expert Rev Mol Diagn (2016) 16(9):933–40. Epub 2016/07/09. 10.1080/14737159.2016.1210513 27388792

[24] Gibb FW McKnight JA Clarke C Strachan MW. Preserved C-Peptide Secretion Is Associated with Fewer Low-Glucose Events and Lower Glucose Variability on Flash Glucose Monitoring in Adults with Type 1 Diabetes. Diabetologia (2020) 63:906–14. 10.1007/s00125-020-05099-3 32034440 PMC7145780

