# Supplementary figures and images for: Comparative Analysis of Islet Auto-Transplantation Outcome Classification Systems: Evaluating Concordance, Feasibility, and a Data-Driven Approach
Source: Transpl Int. 2025 Jul 18;38:14714. doi: 10.3389/ti.2025.14714 (PMC12313549; doi:10.3389/ti.2025.14714)

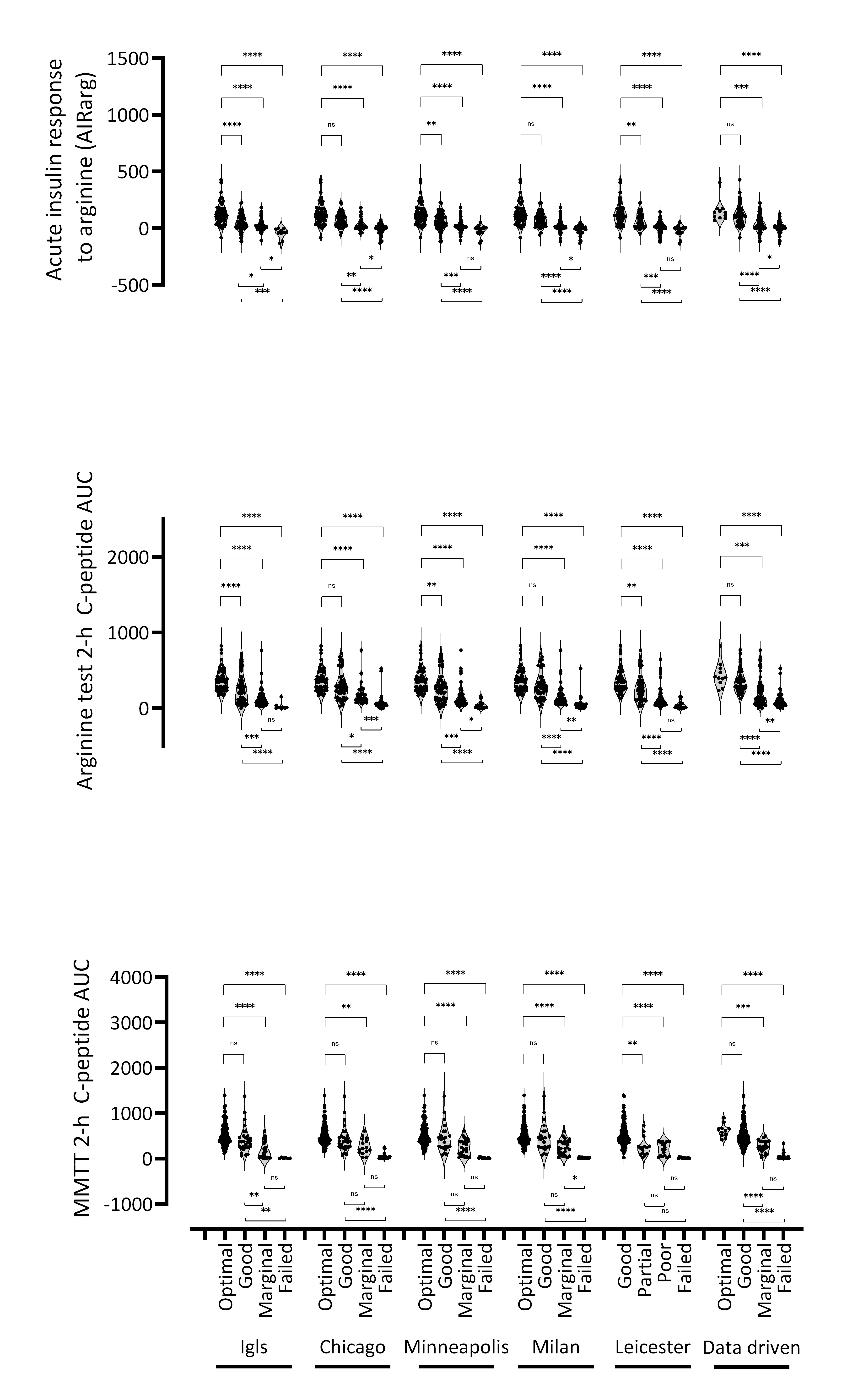

Supplement: Supplementary file 1 [file Image3.jpeg]

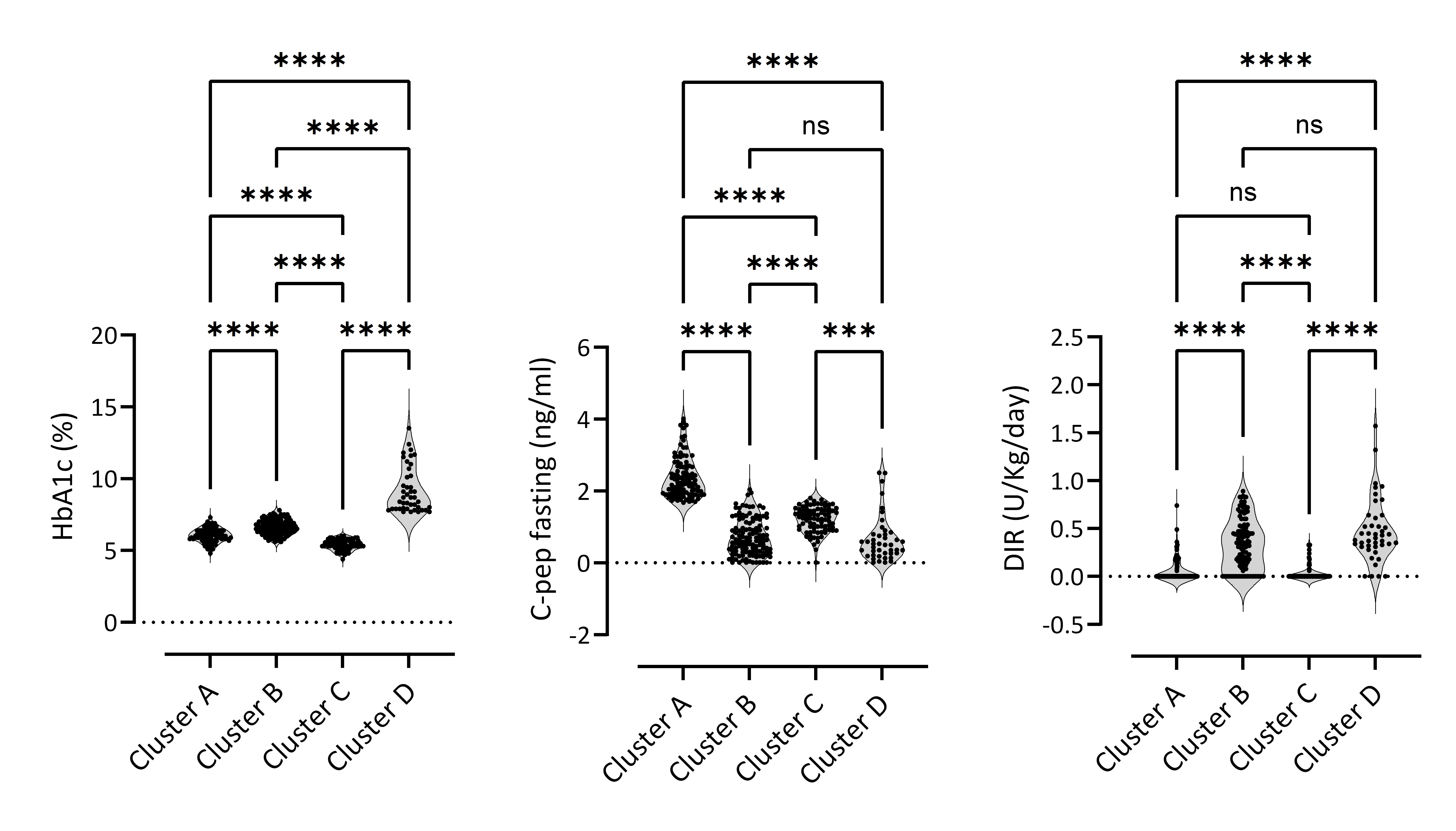

Supplement: Supplementary file 3 [file Image1.jpeg]

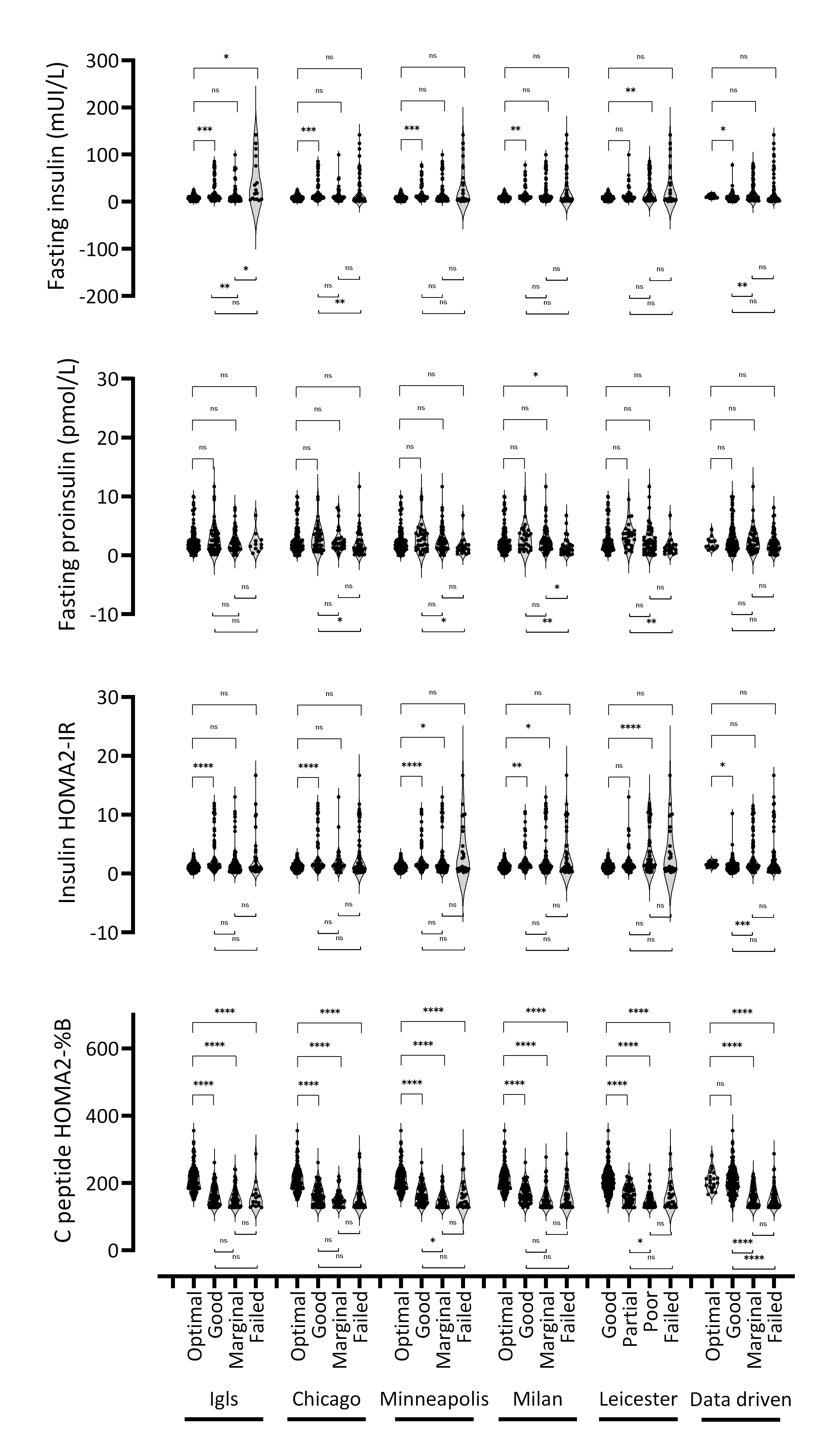

Supplement: Supplementary file 4 [file Image4.jpeg]

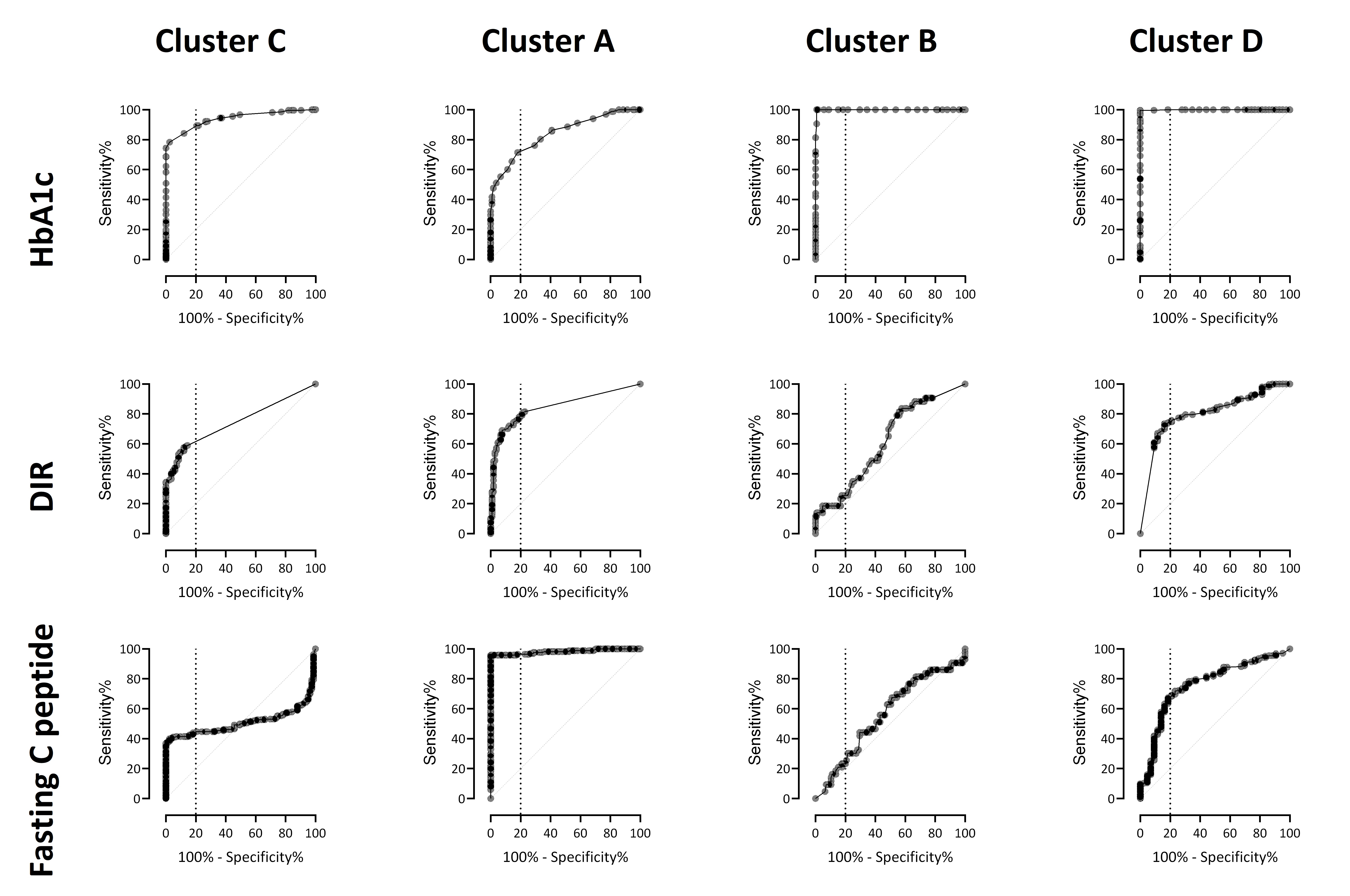

Supplement: Supplementary file 5 [file Image2.jpeg]
