# Supplementary material for: Comparative Analysis of Islet Auto-Transplantation Outcome Classification Systems: Evaluating Concordance, Feasibility, and a Data-Driven Approach
Source: Transpl Int. 2025 Jul 18;38:14714. doi: 10.3389/ti.2025.14714 (PMC12313549; doi:10.3389/ti.2025.14714)
Supplement: Supplementary file 2 [file Table1.docx]

**Supplementary Table 1. Metabolic Parameters by Cluster Classification**

|  | Cluster A | Cluster B | Cluster C | Cluster D |  |
| --- | --- | --- | --- | --- | --- |
| N | 105 | 125 | 83 | 43 | p |
| FPG (mg/dl) | 110 (98-127) | 135 (112-185) | 99 (90-111) | 169 (129-260) | <0.0001^a, b, c, d, f^ |
| Fasting insulin (mUI/L) | 11.1 (8.3-15.1) | 7.4 (4.4-16.4) | 6.3 (4-9.4) | 6.8 (4.6-13) | <0.0001 ^a, b, c, d^ |
| Fasting proinsulin | 2.4 (1.6-34) | 1.7 (0.92.8) | 1.3 (0.7-2.5) | 2 (1.4-3.6) | 0.0006 ^a, b^ |
| HOMA-IR | 1.5 (1.2-2.1) | 1.2 (0.7-3.2) | 0.9 (0.6-1.3) | 1.3 (0.7-2.3) | <0.0001 ^a, b, d, f^ |
| HOMA-beta | 188 (165-231) | 158 (137-185) | 211 (186-234) | 149 (133-165) | <0.0001 ^a, b, c, d, e, f^ |
| Arginine test |  |  |  |  |  |
| Peak C -peptide (ng/ml) | 4.3 (3.6-5.4) | 0.97 (0.5-1.79 | 2.6 (1.9-3.4) | 1.1 (0.7-1.6) | <0.0001^a, b, c, d, f^ |
| 2h C peptide AUC | 113.5 (88-170) | 10.1 (-11-33.3) | 40 (17.4-115) | 21 (8.7-47) | <0.0001 ^a, b, c, d^ |
| Acute insulin response to arginine (AIRarg) | 374 (289-507) | 96 (46-187) | 261 (186-340) | 59 (62-146) | <0.0001^a, b, c, d, f^ |
| MMTT |  |  |  |  |  |
| Peak C -peptide (ng/ml) | 6.7 (5.3-8.8) | 2.9 (1.1-4.5) | 4.7 (4-6.2) | 0.5 (0.2-1.4) | <0.0001^a, b, c, d, f^ |
| Time Peak C -peptide (min) | 90 (60-120) | 90 (52-120) | 90 (60-120) | 120 (90-180) | 0.0166 ^c, e, f^ |
| 2h C peptide AUC | 592 (451-768) | 248 (113-377) | 406 (325-485) | 33.7 (14.1-130) | <0.0001 ^a, b, c, d, f^ |

^a^ Cluster A vs cluster B; ^b^ Cluster A vs Cluster C; ^c^ Cluster A vs Cluster D; ^d^ Cluster B vs Cluster C; ^e^ Cluster B vs Cluster D; ^f^ Cluster C vs Cluster D

**Supplementary Table 2. Comparison of six classification systems used to define graft function after islet autotransplantation: Igls, Chicago, Minnesota, Milan, Leicester, and a Data-Driven model**

| System | Optimal | Good | Marginal | Failed | Notes |
| --- | --- | --- | --- | --- | --- |
| Igls | HbA1c ≤6.5%, no SHE, 0 U/kg/d, any C-peptide | HbA1c <7%, no SHE, any insulin, ≥0.2 ng/mL | HbA1c ≥7%, ≥1 SHE, any insulin, ≥0.1 ng/mL | C-peptide <0.1 ng/mL |  |
| Chicago | HbA1c ≤6.5%, no SHE, 0 U/kg/d, >0.5 ng/mL | HbA1c <7%, no SHE, <0.5 U/kg/d, >0.5 ng/mL | HbA1c ≥7%, ≥1 SHE, ≥0.5 U/kg/d, >0.5 ng/mL | C-peptide ≤0.5 ng/mL |  |
| Minnesota | HbA1c ≤6.5%, no SHE, any insulin, ≥0.2 ng/mL | HbA1c <7%, no SHE, <0.5 U/kg/d, ≥0.2 ng/mL | HbA1c ≥7%, ≥1 SHE, ≥0.5 U/kg/d, ≥0.2 ng/mL | C-peptide <0.2 ng/mL |  |
| Milan | HbA1c ≤6.5%, no SHE, no insulin, >0.5 ng/mL | HbA1c <7%, no SHE, <0.5 U/kg/d, >0.5 ng/mL | HbA1c ≥7%, ≥1 SHE, ≥0.5 U/kg/d, >0.3 ng/mL | C-peptide ≤0.3 ng/mL |  |
| Leicester | No insulin (up to 5 yrs), ≥0.2 ng/mL | <20 U/d, ≥0.2 ng/mL | 20–40 U/d (within 5 yrs), ≥0.2 ng/mL | C-peptide ≤0.5 ng/mL | Focus on insulin use and time post-transplant |
| Data-Driven | Composite score = 12 | Composite score 9 – <12 | Composite score 6 – <9 | Composite score 3 – <6 | Scores assigned by HbA1c, DIR, and C-peptide |

**Supplementary Table 3 Evaluation of the consistency and differentiation capacity of the classification systems based on insulin secretion parameters.**

|  | Acute insulin response to arginine (AIRarg) | | Arginine Test  2h C-peptide AUC | | MMTT  2h C-peptide AUC | |
| --- | --- | --- | --- | --- | --- | --- |
|  |  | CVM (%) |  | CVM (%) |  | CVM (%) |
| Igls | | | | | | |
| Optimal | 112 (59-172) | 48 | 353 (251-453) | 27 | 469 (361-654) | 29 |
| Good | 38 (8-97) | 100 | 208 (83-334) | 60 | 372 (251-497) | 33 |
| Marginal | 12 (0-38) | 111 | 96 (61-158) | 46 | 87 (24-266) | 85 |
| Failed | -38 (-62 - -7) | -60 | 11 (1-20) | 60 | 6 (1-23) | 80 |
| p | <0.0001^a^ | <0.0001^b^ | <0.0001^a^ | <0.0001^b^ | <0.0001^a^ | 0.0717^b^ |
| Chicago | | | | | | |
| Optimal | 112 (59-172) | 48 | 353 (251-453) | 27 | 477 (378-658) | 27 |
| Good | 60 (25-106) | 65 | 266 (137-369) | 45 | 372 (258-498) | 32 |
| Marginal | 16 (3-50) | 101 | 128 (97-186) | 35 | 226 (136-433) | 51 |
| Failed | 0 (-32-14) | - | 50 (34-65) | 30 | 25 (8-54) | 74 |
|  | <0.0001^a^ | <0.0001^b^ | <0.0001^a^ | <0.0001^b^ | <0.0001^a^ | 0.0036 ^b^ |
| Minneapolis | | | | | | |
| Optimal | 112 (59-172) | 48 | 353 (251-453) | 27 | 469 (361-654) | 29 |
| Good | 52 (17-100) | 88 | 233 (336-112) | 50 | 425 (243-603) | 42 |
| Marginal | 13 (0-32) | 101 | 95 (61-172) | 39 | 226 (62-375) | 70 |
| Failed | -1 (2- -39) | -994 | 24 (11-62) | 90 | 10 (2-1) | 88 |
|  | <0.0001^a^ | <0.0001^b^ | <0.0001^a^ | <0.0001^b^ | <0.0001^a^ | 0.0013 ^b^ |
| Milan | | | | | | |
| Optimal | 112 (59-172) | 48 | 353 (251-453) | 27 | 477 (378-658) | 29 |
| Good | 67 (28-111) | 58 | 269 (145-372) | 39 | 425 (243-603) | 42 |
| Marginal | 14 (0-37) | 101 | 107 (70-176) | 39 | 233 (114-374) | 59 |
| Failed | -1 (-36-6) | -761 | 38 (14-52) | 41 | 14 (2-26) | 62 |
|  | <0.0001^a^ | <0.0001^b^ | <0.0001^a^ | <0.0001^b^ | <0.0001^a^ | 0.0004 ^b^ |
| Leicester | | | | | | |
| Optimal | 111 (57-170) | 49 | 346 (257-433) | 25 | 466 (361-649) | 28 |
| Good | 46 (11-100) | 90 | 232 (112-334) | 49 | 242 (112-270) | 51 |
| Marginal | 14 (-6-38) | 159 | 82 (56-139) | 43 | 201 (54-368) | 78 |
| Failed | -1 (-39-2) | -994 | 24 (11-62) | 90 | 10 (2-21) | 88 |
|  | <0.0001^a^ | 0.0005^b^ | <0.0001^a^ | <0.0001^b^ | <0.0001^a^ | 0.0037 ^b^ |
| Data Driven | | | | | | |
| Optimal | 138 (93-173) | 28 | 405 (341-529) | 17 | 618 (511-666) | 12 |
| Good | 101 (41-147) | 48 | 334 (244-417) | 25 | 457 (359-641) | 29 |
| Marginal | 25 (1-81) | 108 | 134 (72-272) | 68 | 258 (359-641) | 45 |
| Failed | 8 (-6-24) | 179 | 76 (43-126) | 48 | 26 (9-49) | 70 |
|  | <0.0001^a^ | 0.088^b^ | <0.0001^a^ | <0.0001^b^ | <0.0001^a^ | <0.0005^b^ |
| Among different classification | | | | | | |
|  | p ^a^ | | p ^a^ | | p ^a^ | |
| Optimal | 0.9535 | | 0.6842 | | 0.4805 | |
| Good | 0.0002^h^ | | <0.0001 ^h^ | | 0.005^i^ | |
| Marginal | 0.2291 | | 0.0015^e,i^ | | 0.1135 | |
| Failed | 0.005 ^m^ | | <0.0001 ^i,m,p,q^ | | 0.0618 | |

^a^ Kruskal-Wallis test; ^b^ Brown-Forsythe test;

Significant at Dunn's multiple comparisons test: ^c^ Igls vs Leichester; ^d^ Chicago vs Milan; ^e^ Chicago vs Leichester; ^f^ Milan vs Leicester; ^g^Igls vs Milan; ^h^ Data driven vs all others; ^i^ Leicester vs Data Driven; ^l^Minneapolis vs Data Driven; ^m^Igls vs Data Driven; ^n^ Igls vs Chicago; ^o^ Chicago vs Minneapolis; ^p^ Milan vs Data Driven; ^q^ Chicago vs Data Driven

**Supplementary Table 4 Evaluation of the consistency and differentiation capacity of the classification systems based on metabolic parameters.**

|  | C peptide  HOMA2-%B | | Insulin  HOMA2-IR | | Fasting  insulin | | Fasting  proinsulin | |
| --- | --- | --- | --- | --- | --- | --- | --- | --- |
|  | % | CVM (%) |  | CVM (%) | mUI/L | CVM (%) | pmol/L | CVM (%) |
| Igls | | | | | | |  |  |
| Optimal | 209 (186-231) | 11 | 1 (0.7-1.6) | 40 | 8 (5-11) | 44 |  |  |
| Good | 156 (138-181) | 13 | 1.5 (1-2.4) | 40 | 10 (7-17) | 41 |  |  |
| Marginal | 146 (131-168) | 12 | 1.2 (0.6-2.1) | 54 | 7 (4-12) | 56 |  |  |
| Failed | 144 (133-168) | 11 | 1 (0.7-1.6) | 45 | 22 (5-92) | 77 |  |  |
| p | <0.0001^a^ | 0.18 ^b^ | <0.0001^a^ | <0.0001^b^ | <0.0001^a^ | <0.0001^b^ |  |  |
| Chicago | | | | | | |  |  |
| Optimal | 206 (186-230) | 10 | 1.1 (0.7-1.6) | 36 | 8 (5-11) | 43 |  |  |
| Good | 160 (139-183) | 13 | 1.5 (1.1-2.4) | 40 | 11 (8-18) | 39 |  |  |
| Marginal | 147 (137-162) | 8 | 1.3 (1-2.2) | 38 | 8 (6-12) | 33 |  |  |
| Failed | 147 (132-178) | 12 | 1 (0.7-4.2) | 60 | 6 (3-23) | 62 |  |  |
|  | <0.0001^a^ | 0.036^b^ | <0.0001^a^ | <0.0001^b^ | 0.0003^a^ | <0.0001^b^ |  |  |
| Minneapolis | | | | | | |  |  |
| Optimal | 209 (186-231) | 11 | 1 (0.7-1.6) | 40 | 8 (5-11) | 44 |  |  |
| Good | 161 (141-185) | 13 | 1.5 (1.1-2.2) | 33 | 10 (8-16) | 38 |  |  |
| Marginal | 144 (130-164) | 10 | 1.3 (0.8-2.4) | 46 | 8 (5-14) | 47 |  |  |
| Failed | 150 (131-183) | 13 | 1 (0.6-7.6) | 70 | 7 (4-45) | 68 |  |  |
|  | <0.0001^a^ | 0.006^b^ | <0.0001^a^ | <0.0001^b^ | 0.0008^a^ | <0.0001^b^ |  |  |
| Milan | | | | | | |  |  |
| Optimal | 206 (186-230) | 10 | 1.1 (0.7-1.6) | 36 | 8 (5-11) | 43 |  |  |
| Good | 164 (144-185) | 12 | 1.5 (1.1-2.2) | 33 | 10 (8-15) | 32 |  |  |
| Marginal | 144 (130-165) | 10 | 1.3 (0.9-2.6) | 46 | 8 (6-15) | 47 |  |  |
| Failed | 152 (133-183) | 14 | 1.1 (0.6-4.8) | 64 | 7 (4-35) | 66 |  |  |
|  | <0.0001^a^ | 0.1^b^ | 0.0005^a^ | <0.0001^b^ | 0.0065^a^ | <0.0001^b^ |  |  |
| Leicester | | | | | | |  |  |
| Optimal | 204 (184-229) | 10 | 1.1 (0.7-1.6) | 36 | 8 (5-11) | 42 |  |  |
| Good | 164 (143-185) | 13 | 1.3 (0.9-1.9) | 38 | 9 (6-13) | 35 |  |  |
| Marginal | 141 (132-159) | 8 | 1.6 (1-5.2) | 50 | 10 (6-35) | 53 |  |  |
| Failed | 150 (131-183) | 13 | 1 (0.6-7.6) | 70 | 7 (4-45) | 68 |  |  |
|  | <0.0001^a^ | <0.0001^b^ | <0.0001^a^ | <0.0001^b^ | 0.0068^a^ | <0.0001^b^ |  |  |
| Data Driven | | | | | | |  |  |
| Optimal | 204 (188-228) | 8 | 1.5 (1.2-2.1) | 23 | 11 (9-15) | 26 |  |  |
| Good | 200 (178-226) | 12 | 1.1 (0.7-1.5) | 36 | 8 (5-11) | 38 |  |  |
| Marginal | 152 (135-178) | 13 | 1.4 (0.9-4.4) | 43 | 10 (6-29) | 46 |  |  |
| Failed | 146 (132-168) | 11 | 1.2 (0.6-3) | 61 | 7 (4-17) | 63 |  |  |
|  | <0.0001^a^ | 0.013^b^ | <0.0001^a^ | <0.0001^b^ | 0.0005^a^ | <0.0001^b^ |  |  |
| Among different classification | | | | | | |  |  |
|  | p ^a^ | | p ^a^ | | p ^a^ | |  |  |
| Optimal | 0.91 | | 0.044^h^ | | 0.035 ^h^ | |  |  |
| Good | <0.0001^h^ | | <0.0001 ^l,m,p,q,^ | | <0.0001 ^l,m,p,q,^ | |  |  |
| Marginal | 0.24 | | 0.09 | | 0.039 | |  |  |
| Failed | 0.99 | | 0.98 | | 0.45 | |  |  |

^a^ Kruskal-Wallis test; ^b^ Brown-Forsythe test;

Significant at Dunn's multiple comparisons test: ^c^ Igls vs Leichester; ^d^ Chicago vs Milan; ^e^ Chicago vs Leichester; ^f^ Milan vs Leicester; ^g^Igls vs Milan; ^h^ Data driven vs all others; ^i^ Leicester vs Data Driven; ^l^Minneapolis vs Data Driven; ^m^Igls vs Data Driven; ^n^ Igls vs Chicago; ^o^ Chicago vs Minneapolis; ^p^ Milan vs Data Driven; ^q^ Chicago vs Data Driven
